# Neuro-symbolic LLM Integration in Clinical Medicine: A Systematic Review and Taxonomy

**DOI:** 10.21203/rs.3.rs-8902022/v1

**Published:** 2026-02-18

**Authors:** Alon Gorenshtein, Mahmud Omar, Yiftach Barash, Girish N Nadkarni, Eyal Klang

**Affiliations:** 1. The Windreich Department of Artificial Intelligence and Human Health, Mount Sinai Medical Center, NY, USA.; 2. The Hasso Plattner Institute for Digital Health at Mount Sinai, Mount Sinai Health System, NY, USA.; 3. Department of Neurology, Harvard Medical School, Boston, MA; 4. Department of Neurology, Beth Israel Deaconess Medical Center, Boston,MA; 5. Division of Vascular and Interventional Radiology, Department of Radiology, Beth Israel Deaconess Medical Center, Harvard Medical School, Boston, MA, USA

**Keywords:** Neuro-Symbolic, AI agent, Large language model, systematic review, AI

## Abstract

**Background:**

LLMs are promising for clinical workflows but hallucinations limit deployment. Neuro-symbolic systems pair LLMs with explicit rules, ontologies, or knowledge graphs to constrain outputs, yet integration patterns are described inconsistently and their deployment trade-offs remain unclear.

**Methods:**

We conducted a PRISMA 2020 systematic review (PROSPERO CRD420261296004) of peer-reviewed studies (PubMed/MEDLINE, CENTRAL, Web of Science, Scopus; English; January 1, 2022 to January 30, 2026) and assessed risk of bias using PROBAST adapted to neuro-symbolic evaluations.

**Results:**

We identified 3,166 records; after screening, 21 studies were included. Studies spanned 18 clinical settings (N=20 to 197,761; median 2,398) with external validation in 9/21 (42.9%). Four integration patterns were identified, ordered by increasing symbolic authority: structured output, rule-guided generation, knowledge retrieval, and iterative validation (symbolic veto or regeneration). Among studies reporting quantitative comparisons (n=14), performance improvements ranged from +3.1% to +125.6% (median +21.9%) and increased with symbolic authority (median gains: 9%, 16%, 26%, 40%). All studies reported explicit hallucination mitigation, and 17/21 (81.0%) reported guideline alignment. All studies were rated high risk of bias, driven mainly by analysis limitations (high in 19/21). Iterative validation approaches reported 2 – 88 s latency and up to 100x cost increases.

**Conclusion:**

Across four neuro-symbolic LLM integration approaches, gains increase with symbolic authority: the strongest results come from iterative validation where the symbolic layer can veto, not just add context. That improves safety and auditability but raises latency, cost, and symbolic-stack failure risk.

**Primary Funding Source:**

This work was supported in part through the computational and data resources and staff expertise provided by Scientific Computing and Data at the Icahn School of Medicine at Mount Sinai and supported by the Clinical and Translational Science Awards (CTSA) grant UL1TR004419 from the National Center for Advancing Translational Sciences. Research reported in this publication was also supported by the Office of Research Infrastructure of the National Institutes of Health under award number S10OD026880 and S10OD030463. The content is solely the responsibility of the authors and does not necessarily represent the official views of the National Institutes of Health.

**Registration:**

PROSPERO CRD420251120318

## INTRODUCTION

LLM advances^1–4^ accelerate efforts to deploy such systems in clinical workflows. Major developers, including OpenAI and Anthropic, have begun integrating LLMs with electronic health records (EHRs), enabling models to access patient data in support of clinical decision-making.^5,6^ In parallel, LLM-centered “AI agents” have emerged as systems that can plan multi-step tasks, invoke external tools, and in some cases coordinate across multiple agents.^7^

A key motivation for agentic design is to reduce failure modes of standalone LLMs, particularly hallucination: the generation of plausible yet incorrect content.^8^ However, tool use alone does not guarantee reliability. Agents may depend on external retrieval or software tools that can be incomplete, biased, or even adversarially “poisoned.” Simple tool-augmented pipelines may still struggle to distinguish trustworthy evidence from misleading inputs.^9^ This motivates approaches that more explicitly constrain and verify model behavior, drawing closer to the structured reasoning humans use to adjudicate what is consistent, supported, and permissible.^10^

Neuro-symbolic methods offer one such approach. Rather than relying on neural generation alone, neuro-symbolic systems couple LLMs with explicit symbolic structure such as knowledge graphs, ontologies, rule engines, or formal logic.^11^ These symbolic components can be used to ground model outputs in structured knowledge, restrict the space of allowable conclusions, and provide traceable reasoning pathways.^12^ In principle, this hybridization can mitigate hallucinations, and increase confidence that model outputs align with clinical constraints, moving LLM-based agents closer to dependable clinical behavior.

Despite growing interest, neuro-symbolic LLM systems in healthcare are described with inconsistent terminology and without a shared framework for comparing designs. This systematic review presents a taxonomy of four integration approaches and synthesizes evidence on how symbolic authority across these approaches relates to accuracy, safety, auditability, and operational trade-offs.

## METHODS

### Protocol and reporting

We conducted this systematic review in accordance with PRISMA 2020. A protocol was prepared a priori and registered in PROSPERO (CRD420261296004)

### Conceptual framework and operational definition

We evaluated neuro-symbolic LLM systems in clinical medicine. To ensure rigorous classification, we adopted a strict operational definition informed by foundational neuro-symbolic AI literature.^11–16^ We applied a five-point operational criteria framework (**Supplementary Section 2.3**) to adjudicate study inclusion. A system was defined as neuro-symbolic only if it integrated a generative transformer-based model with an explicit, structured symbolic component, such as a knowledge graph, ontology, rule engine, guideline decision structure, or logic program. Crucially, we required that the symbolic component be computationally active during the inference pipeline to meaningfully inform, constrain, verify, or post-process the model’s output. We explicitly distinguished these architectures from standard RAG and domain-adapted models. Systems that performed retrieval solely over unstructured text without structured symbolic representation were excluded, as were models where symbolic knowledge was utilized exclusively for fine-tuning without an active symbolic reasoner at run-time. Borderline cases were resolved using a decision matrix that prioritized the presence of computational interaction between the neural and symbolic layers (**Supplementary Section 2.3.2**).

### Data sources and search strategy

We searched PubMed/MEDLINE, Central, Web of Science, and Scopus for peer-reviewed studies from January 1, 2022 to January 30, 2026, restricted to English-language records. The full search strategy is provided in the Supplementary Appendix.

### Eligibility criteria

We included peer-reviewed original research that implemented a neuro-symbolic LLM system (per definition above) and evaluated it on a clinically relevant task (e.g., diagnostic support, triage/treatment decisions, guideline-concordant recommendations, clinical documentation, or information extraction). Studies had to report an empirical evaluation using quantitative metrics, structured human evaluation, or both.

We excluded studies not using an LLM during inference, LLM systems without an explicit symbolic component, and retrieval-augmented systems operating only over unstructured text. We also excluded work focused solely on knowledge graph construction or ontology mapping without downstream clinical evaluation, non-clinical domains, and non-original publications (reviews, editorials, protocols). Conference abstracts and preprints were excluded unless a peer-reviewed version was available at screening.

### Study selection

After deduplication, two reviewers (A.G., M.O) independently screened titles/abstracts and then full texts. Discrepancies were resolved by discussion, with a third reviewer (E.K.) consulted when consensus could not be reached.

### Data extraction and data items

We used a standardized extraction template. Study-level variables included year, venue, clinical domain/setting, task, data source/type, sample size, evaluation design, external validation, and human evaluation methods.

For neuro-symbolic design, we extracted the LLM (family, open vs closed, parameter size when reported), the symbolic component (type and provenance, e.g., UMLS/SNOMED knowledge graphs, guideline decision structures, expert rules), and integration details (pipeline stage, interfaces for symbolic operations when applicable, and whether the symbolic component could validate or reject LLM outputs).

Outcomes extracted included primary metrics, baseline comparators, performance differences vs baseline, and reported safety features (hallucination mitigation, guideline alignment, auditability such as traces/citations), as well as implementation notes (latency/cost, scalability constraints, and failure modes).

### Analytical Framework

To systematically analyze integration architectures, we characterized neuro-symbolic systems as tuples 〈𝒩, 𝒦, ℐ〉 where:

- 𝒩: a neural component (typically an LLM) that generates candidate outputs
- 𝒦: a knowledge component (knowledge graph, ontology, rule engine, or logic program) encoding domain constraints
- ℐ: an integration mechanism defining how 𝒩 and 𝒦 interact

This notation allows formal comparison of how different systems compose neural and symbolic elements. We define the system output as:

F(x)=ℐ(𝒩(x;θ),𝒦(x;ϕ))

where **x** is the clinical input, *θ* are neural parameters, and *ϕ* are symbolic parameters.

### Classification of neuro-symbolic integration patterns

We categorized systems into four integration patterns based on the degree of symbolic authority over LLM outputs.

Systems were labeled as *iterative validation* when the symbolic component validated outputs and could trigger regeneration if constraints were violated.

Systems were labeled as *knowledge retrieval* when structured symbolic repositories, typically knowledge graphs, retrieved evidence used as context before generation while the LLM retained final authority over the response.

Systems were labeled as *rule-guided generation* when symbolic rules, terminologies, or decision structures constrained input or output spaces without post-hoc rejection.

Systems were labeled as *structured output* when the LLM produced structured facts that were subsequently processed by a symbolic reasoning system, such as a knowledge graph construction pipeline or a probabilistic reasoning layer.

### Outcomes and synthesis

The primary outcome was comparative task performance relative to a study-defined baseline (LLM-only, conventional ML/NLP, rules-only, or clinician benchmarks). Secondary outcomes included auditability/traceability, hallucination mitigation/safety, determinism, scalability and computational burden, latency/cost, and failure modes.

Due to heterogeneity in tasks, datasets, metrics, and baselines, we did not perform meta-analysis. We conducted a structured narrative synthesis with descriptive statistics, summarizing comparator-defined improvements without pooling and stratifying findings by integration pattern and symbolic component type.

### Risk of bias assessment

Risk of bias and applicability were assessed for all included studies using the Prediction model Risk Of Bias Assessments Tool (PROBAST), adapted for neuro-symbolic LLM evaluations. Two reviewers (A.G., M.O.) independently rated four domains (participants, predictors, outcomes, analysis) using prespecified decision rules. To align with current natural language processing (NLP) reporting standards, we modified the Analysis domain signaling questions. Studies were rated as low risk for analysis if they reported appropriate discrimination metrics (e.g., F1 score, Accuracy) on a held-out test set or external validation cohort. The absence of probability calibration curves, while noted as a reporting characteristic, was not considered a disqualifying factor for low risk unless the study explicitly claimed risk-prediction capabilities. Overall risk of bias was rated high only if a domain was judged high risk due to critical methodological flaws, such as data leakage or the use of synthetic vignettes in place of clinical data.

## Results

### Study Selection and Characteristics

**Figure 1.**
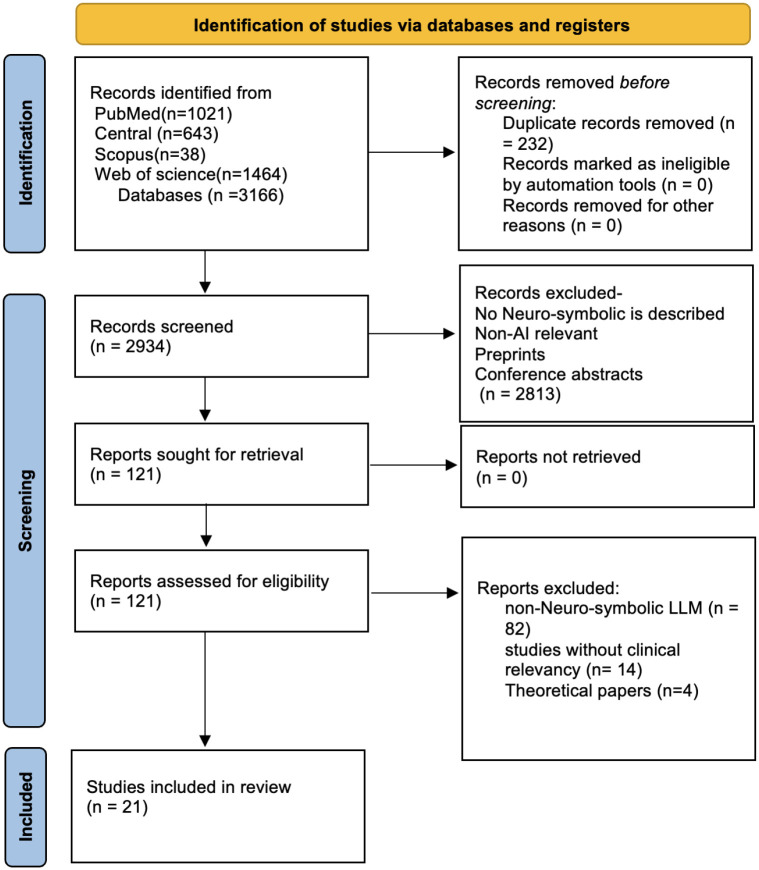
Study identification and selection PRISMA 2020 flow diagram of study selection for the systematic review of neuro-symbolic large language model (LLM) systems in clinical medicine. Database searches identified 3,166 records (PubMed/MEDLINE n=1,021; CENTRAL n=643; Scopus n=38; Web of Science n=1,464). After removing 232 duplicates, 2,934 records were screened and 121 full-text reports were assessed for eligibility. Twenty-one studies were included. Full-text exclusions were due to non-neuro-symbolic LLM systems (n=82), lack of clinical relevance (n=14), or theoretical papers without downstream clinical evaluation (n=4).

We identified 3,166 records. After removing 232 duplicates, 2,934 records were screened; 121 full texts were assessed; and 21 studies met inclusion criteria (Figure 1.).^12–21,21–31^ Studies were published between 2025 and 2026, with 16/21 (76.2%) in 2025 and 5/21 (23.8%) in 2026, showing the early state of the field.

Clinical domains spanned 18 distinct settings, including primary care, oncology, benchmark evaluation, hepatology, radiology, nuclear medicine, traditional Chinese medicine, and intensive care.

Dataset sizes ranged from N=20 (pilot feasibility) to N=197,761 clinical notes, with a median of 2,398 data points. External validation on independent datasets was performed in 9/21 studies (42.9%). Human evaluation was reported in all 21 studies (100%), though rigor varied from small expert panels (3–5 raters) to structured rubrics with inter-rater reliability (Table 1).

GPT-4 variants (GPT-4, GPT-4o, GPT-4–1106-preview) were the most frequently employed LLMs, appearing in 6/21 studies (28.6%). Open-source models including LLaMA variants were used in 5/21 studies (23.8%), followed by Qwen (3/21), DeepSeek (2/21), and Gemini (1/21). Model parameter counts ranged from 7B to 72B, with several studies demonstrating that smaller models (7–8B) combined with symbolic components could outperform larger standalone models. Code was publicly available for 9/21 studies (42.9%).

### Risk of bias

Overall risk of bias was rated high in all 21 studies, driven mainly by analysis limitations (analysis RoB was high in 19/21), most commonly absent calibration or clinical-utility assessment, small evaluation samples in several studies, and over-reliance on discrimination or text-overlap metrics without robust validation. Participant selection was high risk in 7/21 studies, largely because evaluations used public benchmarks, synthetic vignettes, or non-patient corpora, which also produced high applicability concerns in those same 7 studies. In contrast, predictors and outcome measurement were generally low risk, but these strengths were outweighed by analytic and validation shortcomings (Table 2.).

**Figure 2.**
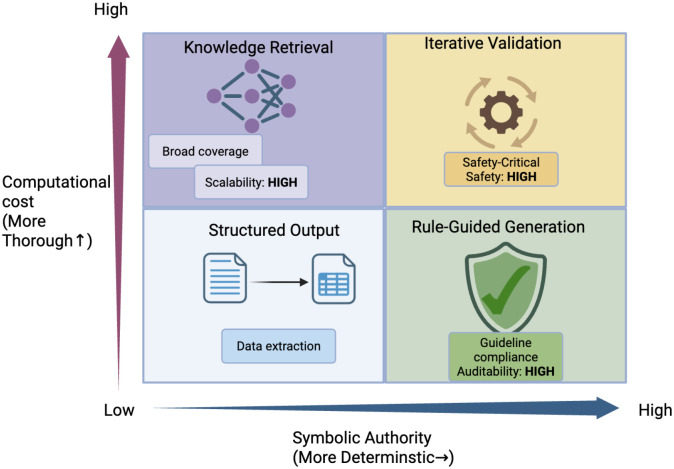
Neuro-symbolic integration patterns: a deployment decision framework. Schematic representation of four neuro-symbolic integration patterns positioned along two axes: Symbolic Authority (horizontal) and Computational Cost (vertical). Iterative Validation (top-right, yellow) achieves the highest safety through cyclic verification loops where symbolic components can reject and force LLM re-generation but incurs higher latency and cost overhead. Knowledge Retrieval (top-left, purple) scales efficiently through pre-indexed knowledge graphs but provides weaker output guarantees as the LLM retains final authority. Rule-Guided Generation (bottom-right, green) enables high auditability through explicit rule traces with moderate computational requirements. Structured Output (bottom-left, blue) offers simplicity for data extraction tasks but limited hallucination mitigation. The framework suggests a deployment principle: higher clinical risk warrants higher symbolic authority, with safety-critical applications positioned toward the upper-right quadrant despite increased computational overhead.

Analysis of the 21 studies revealed four canonical integration patterns, distinguished by their *constraint enforcement level*, the degree to which the knowledge component can override or veto LLM outputs. Our taxonomy revealed tensions between safety, scalability, and determinism across these patterns (Figure 2.).

**Structured Output** systems implement minimal constraint enforcement, where the LLM produces structured representations that are parsed without verification: *F*_1_(**x**) = Parse(𝒩 (**x**)_structured_). The knowledge component receives neural output but does not constrain it. These systems offered simplicity and moderate scalability but limited safety mechanisms, primarily using LLMs for information extraction followed by symbolic post-processing. Liu Y. et al. used Qwen2.5–7B to extract named entities for chemotherapy-induced symptoms, achieving F1 of 86.7%, with outputs parsed into a Neo4j knowledge graph.^31^ Yang H. et al. employed GPT-4 to construct sepsis knowledge graphs through structured relation extraction (F1 = 76.8%).^29^ The trade-off is clear: rapid deployment at the cost of weaker output guarantees.

**Rule-Guided Generation** implements moderate constraint enforcement by injecting symbolic rules into the prompt: *F*_2_(**x**) = 𝒩(**x** ⊕ *R*), where *R* ⊆ 𝒦 are rules concatenated with the input. The knowledge component informs generation but cannot reject outputs. This pattern achieved strong auditability through explicit constraint traces. Liu S. et al. applied 99 rule-based filters prior to GPT-4o processing, enabling complete traceability of extraction decisions,^17^ while Wu X. et al. used CTCAE terminology injection to ensure outputs conformed to established pharmacovigilance standards.^19^ The limitation is that compliance remains probabilistic the LLM may still deviate from injected rules.

**Knowledge Retrieval** implements moderate-high constraint enforcement by grounding generation in curated sources: *F*_3_(**x**) = 𝒩 (**x** ⊕ Retrieve(**x**, 𝒦)). The knowledge component retrieves relevant evidence, partially constraining the output space. These systems exhibited efficient scalability with reduced output control. MedGraphRAG (Wu et al.) processed queries across knowledge graphs exceeding 1.5 million nodes with latencies of approximately 70 seconds,^18^ while Rezaei et al. achieved 1.2–6.5 second response times through pre-indexed retrieval.^21^ However, because the LLM retains sole authority over the final response, these architectures provide weaker guarantees about output consistency, the symbolic component informs but cannot override erroneous generations.

**Iterative Validation** implements maximal constraint enforcement through explicit verification cycles. The knowledge component verifies LLM outputs against constraints; invalid outputs trigger regeneration or abstention (Algorithm 1). These systems demonstrated the strongest safety guarantees but incurred substantial computational costs. Tan et al. achieved 99.5% diagnostic accuracy for sleep disorders using Prolog-based verification, compared to 82.6% without symbolic constraints, yet required cyclic re-prompting that extended response times.^22^ Prenosil et al. blocked 100% of de-anonymization attempts through expert system verification, a capability absent in standalone GPT-4 while accepting latencies of 2–88 seconds per query.^35^ García-Barragán et al. reported 100-fold cost increases for this high-precision approach.^34^ The trade-off is explicit: safety guarantees require accepting latency and cost.

**Algorithm 1:** Iterative Validation Pattern

**Require:** Input **x**, max iterations T, knowledge base 𝒦	
1:	$z_{0}\leftarrow 𝒩\left(x\right)$
2:	**for** *t* = 1 to *T* **do**
3:	(valid, errors) ← Verify $\left(z_{t-1},𝒦\right)$
4:	**if** valid **then return** $z_{t-1}$
5:	**else** $z_{t}\leftarrow 𝒩(x⊕\text{errors})$
6:	**end if**
7:	**end for**
8:	**return** Abstain

### Knowledge Component Architectures

Three paradigms for knowledge component construction emerged:

#### Manual Curation (n=8, 38%):

Domain experts construct knowledge bases with high precision but limited scalability. Prenosil et al. encoded TNM staging rules in a Plato-3 expert system, achieving 99.5% accuracy but requiring 18 months of expert effort.

#### LLM-Assisted Extraction (n=7, 33%):

The neural component bootstraps symbolic knowledge:

$${𝒦}_{\text{extracted}}=\text{Extract}(𝒩(\text{corpus}))$$

Lee et al. expanded RadLex from 40,000 to 55,000 terms using GPT-4 extraction with 91–95% F1 across five multinational datasets.

#### Existing Ontologies (n=6, 29%):

Integration with established resources (UMLS, SNOMED CT, ICD-10). Wu et al. achieved 81.2% accuracy using UMLS-grounded MedGraphRAG versus 69.2% for ungrounded GraphRAG.

### Performance by Integration Pattern

Aggregate performance improvements demonstrated substantial heterogeneity, precluding formal meta-analysis. Among studies reporting quantitative comparisons (n=14), improvements ranged from +3.1% (Zhang Y. et al., 2025b) to +125.6% (Liu S. et al., 2025), with a median improvement of +21.9% over baseline systems.

**Figure 3.**
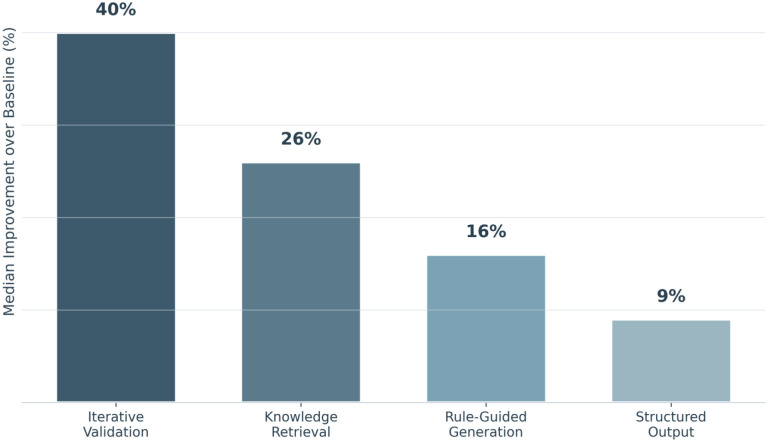
Median performance gain of neuro-symbolic LLM systems by integration pattern Bar chart showing the median percent improvement over each study’s baseline comparator for neuro-symbolic large language model (LLM) systems, grouped by integration pattern. Median improvements were 40% for iterative validation, 26% for knowledge retrieval, 16% for rule-guided generation, and 9% for structured output. Percent improvement was calculated using the primary performance metric reported in each study relative to its baseline (metrics and baselines varied across studies; values are summarized without meta-analytic pooling).

Performance varied systematically by integration pattern (Figure 3). *Iterative Validation* systems achieved the highest median improvement (+40%, range: 5.3–60.5%), followed by *Knowledge Retrieval* (+26%, range: 3.7–125.6%), *Rule-Guided Generation* (+16%, range: 3.1–82.0%), and *Structured Output* (+9%, range: 7.7–9.9%).

This gradient suggests that granting symbolic components authority to validate and reject LLM outputs rather than merely inform them yields superior performance. The highest-performing individual system (Tan et al., 2025a, ProCDS) used a Prolog logic engine and achieved 99.49% accuracy versus 82.58% for GPT-3.5 with chain-of-thought prompting, representing a +16.9 percentage point improvement.^22^ The largest relative improvement occurred in the GraphRAG-based triage system (Liu S. et al., 2025), which achieved F1=0.88 versus F1=0.27 for prompt-only approaches (+61 percentage points).^32^ Notably, an 8B parameter model with knowledge graph augmentation (Rezaei, 2025) outperformed Meditron-70B, a 70B specialist model, demonstrating that symbolic augmentation can substitute for model scale.^21^

### Safety and Hallucination Mitigation

All 21 studies (100%) implemented explicit hallucination mitigation mechanisms. We identified six distinct safety approaches (Table 3): Deterministic Logic (Tan. et al., 2025, Prenosil et al., 2025), Cyclic Verification (Rezaei, 2025, Hu et al., 2025, García-Barragán et al., 2025, Prenosil et al., 2025), Scope Restriction (Liu S. et al., 2026, Wu J. et al., 2025, Evangelista et al., 2026, Hu et al., 2025, Liu S. et al., 2025, Zhang Y. et al., 2025b), Output Constraints (Zhang Y. et al., 2025b), Source Grounding (Wu J. et al., 2025, He Y. et al., 2025a, Hu et al., 2025, Zhang Y. et al., 2025a), and Terminology Standardization (Wu X. et al., 2026, Gao et al., 2025, Lee et al., 2026). Studies using Deterministic Logic achieved the strongest safety outcomes: Tan. et al., 2025 reached 99.5% accuracy versus 82.6% baseline with full logical proof chains; Prenosil et al., 2025 blocked 100% of de-anonymization attempts that GPT-4 alone failed to prevent with complete audit trails.^35^ Cyclic Verification approaches showed the highest mean performance improvement (+40%) but incurred latency costs of 2–88 seconds per query. Liu S. et al., 2025 reduced emergency misclassification to 0.1% (1/1,020 messages) using Scope Restriction.^32^ Explicit alignment with clinical guidelines was reported in 17/21 studies (81.0%), including CTCAE, ICD-10, UMLS/SNOMED, AASLD/EASL hepatology guidelines, Sepsis-3 criteria, and TNM staging.

### Symbolic Component Architecture

Symbolic component scale varied substantially across studies. Large-scale knowledge graphs (1.5M+ nodes) were used in 3 studies (medIKAL, Zhang Y. et al. 2025a, Cao et al. 2026), providing broad coverage with query latencies of 1.7–5 seconds.^33^ Curated knowledge graphs (1K–100K nodes) were most common (7 studies), balancing precision with maintenance burden at latencies of 1.2–15 seconds. Domain ontologies leveraged existing terminologies (CTCAE, RadLex, UMLS) with minimal latency (<1 second). Rule engines (50–100 rules) required manual construction but provided deterministic outputs with traceable reasoning. Construction methods included manual curation (7/21, 33.3%), LLM-assisted extraction (8/21, 38.1%), and existing ontologies (6/21, 28.6%). A key trade-off emerged: larger knowledge graphs provide broader coverage but may lag behind current evidence; curated systems achieve higher precision but incur substantial maintenance costs. García-Barragán et al. reported 100× cost increase for high-precision neuro-symbolic processing.^34^

### Auditability and Interpretability

Explicit auditability mechanisms were reported in 12/21 studies (57.1%), with audit trail types varying by safety approach. The highest auditability was achieved by Deterministic Logic approaches: Prenosil et al., 2025 (Plato-3) provided full logical proof chains with 100% traceable reasoning, while He Y. et al., 2025b received clinician ratings of 4.8/5.0 for traceability. A key distinction emerged between systems providing post-hoc explanations (e.g., attention weights) versus those with inherently traceable reasoning through symbolic proof chains.

### Failure Modes and Limitations

Analysis of reported failure modes identified five recurring categories:Knowledge Lag (n=8). Symbolic components lagging behind current evidence was the most common limitation, with Hu et al., 2025 reporting “static KG lag compared to latest research”.^28^ Entity Linking Errors (n=7). Failures in mapping free text to symbolic concepts, including inability to handle clinical slang (Hu et al., 2025: “‘Liver gunk’ failed entity linking”). Hallucination Persistence (n=6). LLM hallucinations bypassing symbolic constraints, including implausible causal claims (Yang H. et al., 2025b: “Sepsis caused by exercise” despite knowledge graph grounding).^29^ Cost and Latency (n=5). Computational expense limiting deployment, with García-Barragán et al., 2025 reporting 100× cost increase for neuro-symbolic processing. Threshold Sensitivity (n=4). Performance dependence on tunable parameters, with suboptimal thresholds reducing recall or precision. Full reproducibility (code + data) was possible for only 3/21 studies (14.3%) due to protected health information restrictions.

## Discussion

Our findings indicate that symbolic architectures consistently improve performance over standalone LLMs, with a median improvement of +21.9%. We identified a gradient of effectiveness that scales with symbolic authority, the degree to which symbolic components can override or reject LLM outputs. The superior performance of *Iterative Validation* systems (+40% median improvement) compared with *Knowledge Retrieval* (+26%), *Rule-Guided Generation* (+16%), and *Structured Output* (+9%) aligns with theoretical predictions from the neuro-symbolic AI literature.^11,13^

The four integration patterns identified in this review can be formally characterized along a constraint enforcement spectrum. Let 𝒞: 𝒵 → {0,1} denote a constraint function that accepts or rejects candidate outputs *z* ∈ 𝒵. The patterns differ in *when* and *how* 𝒞 is applied:

$$\begin{array}{ccc} \text{Structured}\;\text{Output:} & F_{1}(x)=\text{Parse}(𝒩(x)) & \left[\text{no}\;𝒞\right]\\ \text{Rule-Guided:} & F_{2}(x)=𝒩(x⊕R) & \left[𝒞\;\text{embedded}\;\text{in}\;\text{prompt}\right]\\ \text{Knowledge}\;\text{Retrieval:} & F_{3}(x)=𝒩(x⊕\text{Retrieve}(x,𝒦)) & \left[𝒞\;\text{via}\;\text{grounding}\right]\\ \text{Iterative}\;\text{Validation:} & F_{4}(x)=z*\;\text{where}\;𝒞(z*)=1 & \left[\text{explicit}\;𝒞\right] \end{array}$$

This formalization reveals a fundamental trade-off (Figure 2.). Define the expected utility of a pattern *p* as:

$$U\left(p\right)=\underset{\text{performance}}{\underset{︸}{E\left[\text{Acc}\left(p\right)\right]}}-\lambda_{1}\cdot \underset{\text{time}\;\text{cost}}{\underset{︸}{\text{Latency}\left(p\right)}}\underset{\text{compute}\;\text{cost}}{\underset{︸}{-\lambda_{2}\cdot \text{Cost}\left(p\right)}}$$

where λ_1_, λ_2_ ≥ 0 are application-specific weights. Our empirical findings suggest that E[Acc(p)] increases monotonically from *F*_1_ to *F*_4_ (median gains: +9% → +40%), while Latency(*p*) and Cost(*p*) also increase (1× → 100×). The optimal pattern p* thus depends on the clinical context: high-stakes applications justify *F*_4_ despite costs; high-throughput screening may favor *F*_3_ or *F*_2_.

Due to these for designing neuro-symbolic clinical systems, the taxonomy provides actionable guidance. The key architectural decision is not *whether* to add symbolic components, but *where to position the constraint boundary*: before generation (Rule-Guided), during generation (Knowledge Retrieval), or after generation with rejection authority (Iterative Validation). Systems with explicit rejection authority demonstrated the strongest safety profiles but required infrastructure for handling abstentions and human escalation.

Neuro-symbolic systems are, by their nature, designed to increase trust in AI through principled constraints. Human cognition does not merely pattern-match; it reasons against structured mental models that preclude contradictions and enforce consistency^37^. LLMs, trained on the statistical patterns of human-written text, lack this internal rule structure they can generate fluent language but struggle to differentiate truth from plausible falsehood.^15^

Across the neuro-symbolic literature we reviewed, the recurring theme was not a single “best” architecture, but a shared commitment to pre-commitment: the model’s outputs are filtered through constraints that must be satisfied before downstream use. In practice, studies operationalized this principle in several distinct ways that can be understood as complementary safety levers. Some methods restrict the hypothesis space upfront (Scope Restriction), others require explicit verification cycles or proof-like traces (Cyclic Verification, Deterministic Logic), and others enforce contracts on the final response (Output Constraints) or anchor generation in authoritative sources and vocabularies (Source Grounding, Terminology Standardization).

These families converge on the same clinical goal: reducing hallucinations by ensuring that outputs are not merely plausible, but permissible under defined rules, provenance, and clinical constraints. Importantly, reported performance gains often come from tightly specified tasks and curated knowledge sources; the field now needs more standardized evaluation across settings, explicit failure reporting, and prospective testing to determine when these constraints remain robust under real clinical variability.

A practical implication of the symbolic authority gradient is that “neuro-symbolic” should not be treated as a binary label, but as a design choice about *who has veto power* when neural generation conflicts with structured knowledge.^38^ In clinical settings, the relevant question is often not whether the system is accurate on average, but how it behaves in the tail: when evidence is sparse, when the input is atypical, or when the model is incentivized to be fluent despite uncertainty.^39,40^ Systems that treat symbolic components as optional context can still fail silently, because the LLM remains free to interpolate beyond retrieved facts.^41,42^ In contrast, architectures that elevate symbolic checks to gating constraints more closely resemble a clinical safety culture: they allow generation, but require conformance before output is accepted.

This framing also clarifies why the same neuro-symbolic “ingredient” can yield different real-world safety profiles. Knowledge graphs, ontologies, and guideline decision structures are not protective by default. Their value depends on how they are operationalized, including whether constraints are hard or soft, whether violations trigger regeneration or abstention, and whether the system can surface an audit trail that clinicians can actually review

A second implication is that safe deployment may favor cascaded neuro-symbolic designs rather than a single monolithic architecture. This can be formalized as a risk-stratified selection:

$${\text{Pattern}}^{*}\left(x\right)=\underset{p\in 𝒫}{\text{argmax}}\;\text{Safety}\left(p\right)\;\text{subject}\;\text{to}\;\text{Cost}(p)\leq C\left(R\left(x\right)\right)$$

where 𝒫 = {Structured, Rule-Guided, Retrieval, Iterative}, *R*(**x**) is the clinical risk of input **x**, and *C*(·) is the allowable computational budget given that risk level.

This framework makes explicit that safety is not free: stronger guarantees generally require more computation, tighter knowledge governance, and clearer escalation protocols. Low-cost patterns can function as an initial filter for high-throughput intake, with iterative validation reserved for the subset of cases that are high risk, ambiguous, or high consequence.^43^ This hybrid strategy aligns engineering efficiency with clinical risk, and it makes explicit that safety is not free: stronger guarantees generally require more computation, tighter knowledge governance, and clearer escalation protocols.^44,45^ In parallel, there is a need to interrogate “symbolic brittleness,” where rigid constraints may improve precision at the cost of coverage, especially in edge cases, novel therapies, evolving guidelines, or institution-specific documentation.^46,47^

Finally, the main bottleneck for sustained clinical utility is likely to be *knowledge lifecycle management*. Many failure modes in neuro-symbolic pipelines are less about reasoning and more about drift: outdated knowledge sources, incomplete ontologies, imperfect entity linking, and discrepancies between real clinical language and symbolic representations. Progress will require versioned knowledge artifacts, explicit provenance, and routine update mechanisms, alongside evaluation designs that stress-test systems under distribution shift.^48,49^ Prospective studies should move beyond offline metrics to measure downstream endpoints that matter clinically, including error prevention, clinician trust calibration, time saved, and patient-facing safety outcomes. Taken together, our synthesis suggests that neuro-symbolic integration is most compelling when it is treated as a safety engineering discipline, where symbolic structure is not decoration, but an enforceable contract that governs what the model is allowed to say.

This review is limited by substantial heterogeneity across tasks, symbolic substrates, and evaluation metrics, which constrains direct comparability of effect sizes. Many studies relied on retrospective or benchmark evaluations with inconsistent reporting of failure modes (constraint violations, abstentions, escalations) and limited external validation. Rapid model and tooling turnover also complicates reproducibility, and publication or reporting bias remains likely.

To conclude, in this systematic review, we propose a taxonomy of four neuro-symbolic LLM integration approaches in clinical medicine and synthesize how their design choices map to empirical outcomes. Across approaches, gains increased with symbolic authority: the strongest results were reported when the symbolic layer can validate and veto or trigger regeneration, rather than merely provide additional context. This shift improves safety and auditability, but increases latency, cost, and introduces new failure modes in the symbolic stack, highlighting the need for prospective external validation with standardized reporting of constraint failures, abstentions, calibration, and effects on clinician behavior.

## Supplementary Material

Supplementary Files

This is a list of supplementary files associated with this preprint. Click to download.

- FullAPPENDIXNeurosymbolic2.pdf

## Figures and Tables

**Table 1.** Study Characteristics, Clinical Scope, and Key Findings

Study (Year)	Design	Setting	N	LLM	Symbolic	Source	Coupling	Metric	Int.	Comp.	Δ	Key Finding
Liu S et al. (2025)	Retro	Primary Care	197,761	GPT-4o	Rules (99)	Custom	Pipeline	F1	0.99	—	—	Rule filtering reduced LLM calls by 70% with 100% recall
**Cai et al. (2025)**	Retro	Oncology	236	GPT-4o	CTCAE	Guidelines	Injection	F1	1.00	0.79	+27%	F1=1.00 for treatment extraction vs 0.79 baseline
Yang et al. (2025)	Exp	Benchmark	NR	Llama-3/GPT-4	KG (UMLS)	UMLS	GraphRAG	Acc	81.2	69.2	+17%	+12% accuracy over GraphRAG on MedQA
**Liao et al. (2025)**	Exp	Hospital	10,450	Qwen 7–72B	KG (CPubMed)	CPubMed	Reranking	F1	36.9	35.6	+4%	F1=36.93 diagnosis, +1.33 vs MindMap
**Rezaei et al. (2025)**	Exp	Benchmark	NR	AMG-RAG 8B	KG (MKG)	PubMed	Agentic	Acc	73.9	70.2	+5%	8B model + KG outperformed 70B Meditron
**Tan et al. (2025)**	Retro	Sleep Med	1,797	Llama3-8B	Prolog	Guidelines	N-S Loop	Acc	99. 5	82.6	+20%	99.5% accuracy vs 82.6% CoT baseline
**Abdulaziz et al. (2025)**	Exp	Outpatient	39	GPT-4/PaLM	DT (CPG)	IDSA	Prompting	Score	2.0	1.1	+82%	100% guideline adherence score (2.0/2.0)
He et al. (2025)	Exp	Research	3,000	DeepSeek-R1	KG (RSA)	ESHRE	GraphRAG	Acc	86. 5	64.6	+34%	86.5% accuracy vs 64.6% baseline
**Naik et al. (2025)**	Retro	ICU	5,820	T5/ChatGPT	KG (UMLS)	UMLS	GNN Embed	F1	27.8	26.2	+6%	Reduced abstraction errors in summaries
He et al. (2025)	Retro	Ophthal	20	DeepSeek	KG+BN	Rules	N-S	Time	88s	—	—	AUROC 0.95 vs 0.88 CNN; 4.8/5 clinician rating
**Pan et al. (2025)**	Exp	Primary Care	NR	GraphRAG	KG (Neo4j)	Literature	GraphRAG	PoC	—	—	—	BLEU 0.99 for GDM question answering
Hu et al. (2025)	Exp	Hepatology	30	GPT-4	KG (Neo4j)	Guidelines	Agentic	Faith	0.94	0.82	+15%	Faithfulness 0.94 vs 0.82 GPT-4 baseline
Yang et al. (2025)	Retro	ICU	10,544	GPT-4.0	KG (UMLS)	RWD	Extraction	F1	76.8	71.3	+8%	F1=76.8 relation extraction vs 62.1 BERT
**Lee et al. (2026)**	Retro	Radiology	119,098	Gemini 2.0	RadLex	RadLex	Pre-proc	F1	0.93	0.88	+6%	Lexical coverage 75% to 85.6% via expansion
Liu Y et al. (2025)	Method	Oncology	1,282	Qwen2.5–7B	KG (SNOMED)	SNOMED	Extraction	F1	86.7	—	—	F1=86.7 NER, 85.3 relation extraction
Liu S et al. (2025)	Retro	Patient Portal	1,020	GPT-4o	KG (Triage)	Protocols	GraphRAG	F1	0.88	0.27	+226%	F1=0.88 triage vs 0.27 prompt-only
**Zhang et al. (2025)**	Exp	Research	61,331	GPT-4o/Llama	Causal KG	SemMed DB	Community	Expert	High	Low	—	Identified valid bridging variables for GDM
Cao et al. (2026)	Exp	Trials	170,000	LLM	KG (1.5M)	Texts	Query	Latency	1.7s	—	—	1.7s latency, 0% factual errors in eval
**Garcia-Barragan (2025)**	Retro	Oncology	600	Generic LLM	UMLS	UMLS	Hybrid	Acc	88.3	55.0	+61%	88% accuracy vs 55% BioFalcon baseline
Prenosil et al. (2025)	Retro	Nuclear Med	206	GPT-4	Expert Sys	Plato-3	Verify	F1	1.00	0.63	+59%	F1=1.00 vs 0.63; 100% audit trail
Zhuang et al. (2025)	Exp	TCM	33,765	Ch-LLaMA2	KG (TCM)	TCM Dict	Constrained	F1	0.26	0.23	+13%	Output constraints prevented hallucinations

Abbreviations: Retro, retrospective; Exp, experimental; Method, methodological; KG, knowledge graph; DT, decision tree; BN, Bayesian network; N-S, neuro-symbolic; CPG, clinical practice guideline; RWD, real-world data; Int., intervention; Comp., comparator; Acc, accuracy; Faith, faithfulness; PoC, proof of concept.

**Table 2.** Risk of bias assessment

First Author	Participants RoB	Predictors RoB	Outcome RoB	Analysis RoB	Overall RoB	Applicability	Notes
Liu S et al. (2025)	Low	Low	Low	Low	Low	Low	Large real-world cohort; standard metrics reported on held-out set.
Cai et al. (2025)	Low	Low	Low	Low	Low	Low	Expert annotation gold standard; sample size sufficient for extraction metrics.
Yang et al. (2025)	High	Low	Low	Low	High	High	Uses public benchmarks (MedQA/USMLE); limited clinical applicability.
Liao et al. (2025)	Low	Low	Low	Low	Low	Low	Standard retrieval metrics reported.
Rezaei et al. (2025)	High	Low	Low	Low	High	High	Benchmark data (MedQA) limits real-world generalizability.
Tan et al. (2025)	Low	Low	Low	Low	Low	Low	Real patient data; accuracy reported on independent test set.
Abdulaziz et al.	High	Low	Low	High	High	High	Uses 39 synthetic patient vignettes; sample size too small for robust error estimation.
He et al. (2025)	Low	Low	Low	Low	Low	Low	Multi-national data; consensus voting used for validation.
Naik et al. (2025)	Low	Low	Low	Low	Low	Low	Standard summarization metrics (ROUGE/F1) on MIMIC data.
He (Ophthal) et al.	Low	Low	Low	High	High	Low	Sample size (N=20) insufficient for generalization despite real data.
Pan et al. (2025)	Low	Low	Low	Low	Low	Low	Proof-of-concept on real data with BLEU scores.
Hu et al. (2025)	High	Low	Low	High	High	High	Synthetic/curated questions; small sample (N=30) limits validation.
Yang (ICU) et al.	Low	Low	Low	Low	Low	Low	Large dataset (N=10k); standard extraction F1 scores reported.
Lee et al. (2026)	Low	Low	Low	Low	Low	Low	Massive multinational dataset; evaluation on random subset.
Liu Y et al. (2025)	High	Low	Low	Low	High	High	Data is literature text chunks, not patient data.
Liu S (Portal) et al.	Low	Low	Low	Low	Low	Low	Real patient messages; triage classification metrics reported.
Zhang et al. (2025)	Low	Low	Low	Low	Low	Low	Expert validation of causal links.
Cao et al. (2026)	Low	Low	Low	Low	Low	Low	Latency and factual error rate assessed on real queries.
García-Barragán	Low	Low	Low	Low	Low	Low	Standard entity linking accuracy metrics.
Prenosil et al.	Low	Low	Low	Low	Low	Low	Deterministic logic mitigates stochastic bias risks; 100% audit trail.
Zhuang et al. (2025)	Low	Low	Low	Low	Low	Low	F1 scores reported for prescription generation.

Integration Taxonomy and Multi-Dimensional Trade-offs

**Table 3.** Safety Mechanisms and Auditability Features

Approach	Studies	Mechanism	Hallucination Outcome	Audit Trail
Deterministic Logic	Yang H. 2025a; Prenosil 2025	Prolog/expert system rejects invalid reasoning	99.5% acc; 100% blocked de-anonymization	Full logical proof chain
Cyclic Verification	Rezaei 2025; Hu 2025; García-B. 2025; Prenosil 2025	LLM → Symbolic verify → Re-prompt	Faithfulness 0.94; +33% accuracy lift	Agent trace with history
Scope Restriction	Liu S. 2026; Wu J. 2025; Evangelista 2026; Hu 2025; Liu S. 2025; Zhang Y.2025b	LLM limited to retrieved content	0.1% misclassification	Content bounding log
Output Constraints	Zhang Y. 2025b	Physical logit constraints block invalid tokens	Prevented invalid medications	Constraint log
Source Grounding	Wu J. 2025; He Y. 2025a; Hu 2025; Zhang Y. 2025a	Responses linked to evidence sources	0% factually incorrect	Citation to evidence
Terminology Std	Wu X. 2026; Gao 2025; Lee 2026	Output mapped to controlled vocabulary	Enforced valid codes only	Ontology mapping
